# Nanostructured Lipid Carriers for Sustained Release and Enhanced Delivery of *Vanda coerulea* Protocorm Extract

**DOI:** 10.3390/pharmaceutics17081076

**Published:** 2025-08-20

**Authors:** Piyatida Amnuaykan, Pimporn Anantaworasakul, Kodpaka Lueadnakrob, Pongsagon Kunkul, Wilasinee Chokrungsarid, Aiya Thummanuwong, Saranya Juntrapirom, Watchara Kanjanakawinkul, Wantida Chaiyana

**Affiliations:** 1 Department of Pharmaceutical Sciences, Faculty of Pharmacy, Chiang Mai University, Chiang Mai 50200, Thailand; piyatida_am@cmu.ac.th (P.A.); pimporn.a@cmu.ac.th (P.A.); kodpaka_luead@cmu.ac.th (K.L.); pongsagon_k@cmu.ac.th (P.K.); wilasinee_ch@cmu.ac.th (W.C.); aiya_t@cmu.ac.th (A.T.); 2 Chulabhorn Royal Pharmaceutical Manufacturing Facilities by Chulabhorn Royal Academy, Chon Buri 20180, Thailand; saranya.jun@cra.ac.th (S.J.); watchara.kan@cra.ac.th (W.K.); 3 Center of Excellent in Pharmaceutical Nanotechnology, Faculty of Pharmacy, Chiang Mai University, Chiang Mai 50200, Thailand; 4 Research Center of Deep Technology in Beekeeping and Bee Products for Sustainable Development Goals (SMART BEE SDGs), Chiang Mai University, Chiang Mai 50200, Thailand; 5 Multidisciplinary and Interdisciplinary School, Chiang Mai University, Chiang Mai 50200, Thailand

**Keywords:** Blue Vanda, orchid, catechin, nanostructured lipid carriers, sustained release, skin retention

## Abstract

**Background/Objectives:** This study aimed to develop a nanostructured lipid carrier (NLC) system incorporating a catechin-rich *Vanda coerulea* extract for topical cosmetic applications and to evaluate its physicochemical properties, release behavior, and skin retention performance. **Methods:** Blank NLCs were prepared using hot emulsification followed by sonication, with glyceryl monostearate, caprylic triglyceride, Poloxamer^®^ 188, and Tween^®^ 80 as the formulation components. NLCs with varying solid-to-liquid lipid ratios were developed while maintaining a constant total lipid content of 5% *w*/*w*. The formulations were characterized based on their particle size, polydispersity index (PDI), zeta potential, and physical stability, including stability after a heating–cooling cycle test. The effect of ultrasonication duration was also evaluated. The optimized NLC was then loaded with a *V. coerulea* extract and evaluated for in vitro release and skin retention using catechin as a marker. **Results:** The NLC with a particle size of 235.5 ± 29.8 nm, a narrow PDI range of 0.382 ± 0.090, and a strong zeta potential of −29.8 ± 0.3 mV was selected for the incorporation of the *V. coerulea* extract. The extract-loaded NLC exhibited a sustained release over 24 h, significantly different from the *V. coerulea* extract solution (*p* < 0.05). Skin retention studies revealed that the NLC achieved approximately twice the catechin retention compared to the solution at the 1 h time point (1.30 ± 0.01% vs. 0.68 ± 0.03% *w*/*w*). **Conclusions:** The *V. coerulea*-extract-loaded NLC demonstrated favorable physicochemical properties, sustained release behavior, and enhanced skin retention. These findings support its potential as a promising topical delivery system for antioxidant-rich botanical extracts in cosmetic applications.

## 1. Introduction

The Orchidaceae family is the largest among flowering plants, encompassing over 35,000 species across approximately 850 genera [1]. Among them, Vanda species are particularly notable, not only for their ornamental appeal but also for their traditional medicinal uses and promising pharmacological properties [2]. The Vanda genus, comprising around 73 species primarily found in Southeast Asia, faces population decline due to extensive use, leading to the classification of some species as threatened [2]. *Vanda coerulea* Griff. ex Lindl. is a widely admired orchid species due to its striking beauty. Although it is not currently classified as threatened, it is challenging to cultivate through conventional methods. Therefore, cell culture techniques offer a more efficient alternative for its propagation. Our previous study demonstrated the successful use of elicitor-treated protocorm cultures of *V. coerulea* to enhance the production of bioactive compounds with significant biological activities relevant to cosmeceutical applications [3]. Protocorms developed through callus induction and treated with chitosan not only exhibited distinct morphological changes but also showed elevated levels of key phytochemicals, including catechin, syringic acid, rutin, ellagic acid, rosmarinic acid, and quercetin [3]. Notably, the methanolic extract of chitosan-treated *V. coerulea* protocorms demonstrated remarkable biological properties relevant to the cosmeceutical applications, including potent collagenase inhibition, anti-tyrosinase activity, and strong anti-inflammatory effects, and exhibited no signs of irritation or cytotoxicity [3], highlighting the promise of this biotechnological approach for the sustainable production of valuable cosmeceutical active ingredients.

Cosmeceuticals are scientifically formulated topical products containing active ingredients that target specific skin concerns at the cellular level and are supported by evidence-based research to ensure their efficacy and safety [4]. Therefore, achieving optimal skin benefits from cosmeceutical products depends not only on the incorporation of effective and safe active ingredients but also on efficient delivery systems that ensure their stability, bioavailability, and targeted action within the skin. The hydrophilic nature of a methanolic extract of *V. coerulea* protocorms poses challenges for efficient skin permeation. Therefore, the use of appropriate nanodelivery systems would be essential to enhance the skin penetration, stability, and bioavailability of this type of extract, thereby maximizing its efficacy in cosmeceutical applications.

Among the various nanodelivery systems, lipid-based nanocarriers are of interest for enhancing the skin penetration of hydrophilic extracts. These nanocarriers, such as nanoemulsions, liposomes, and lipid nanoparticles, enhance the delivery of hydrophilic compounds through the skin barrier by interacting with skin lipids to increase permeability and facilitate deeper penetration into the skin layers [5]. Catechin-loaded nanocarriers offer several advantages for topical application, including a high loading capacity, controlled and sustained release, enhanced catechin stability, improved skin absorption, and targeted delivery to specific sites of action [6]. Within the category of lipid-based nanocarriers, solid lipid nanoparticles (SLNs) and nanostructured lipid carriers (NLCs) represent versatile delivery systems that merge the advantages of colloidal lipid emulsions with those of solid matrix particles, offering considerable benefits for topical and transdermal skin applications [7,8]. These lipid nanoparticles possess favorable properties for topical application, including strong adhesion to the stratum corneum and enhanced drug penetration into viable skin layers, which are attributed to their occlusive effect through film formation when the particle sizes range between 200 and 400 nm [9]. However, NLCs feature a distinctive matrix composed of both solid and liquid lipids with varying melting points, resulting in an imperfect crystalline structure that sets them apart from SLNs [10]. The nanostructured design of NLCs, which incorporates both solid and liquid lipids, can be tailored to suit specific drug delivery requirements, offering a greater drug loading capacity and improved stability compared to SLNs [11]. The active ingredients can be either dissolved or finely dispersed within the lipid matrix of NLCs [12]. Surfactants are commonly incorporated to stabilize the nanoparticles and prevent aggregation, enabling effective encapsulation of hydrophobic substances, with the loading capacity influenced by the lipid solubility of the ingredients and their distribution within the solidified lipid matrix [13,14,15]. Additionally, lipid nanoparticles have emerged as a highly effective delivery mechanism in cosmetic applications because they provide sustained release, protect sensitive bioactive compounds, and facilitate skin penetration [16,17]. A previous study suggested that NLCs could achieve a significantly higher encapsulation efficiency of 90% for catechins, particularly (−)-epigallocatechin gallate (EGCG), compared to SLNs, while also exhibiting high storage stability, limited release of the active compound, and low toxicity [18].

Accordingly, this research aimed to develop an NLC-based delivery system for a *V. coerulea* protocorm extract, evaluating its skin delivery performance to ensure efficient targeting of specific skin layers for potential cosmeceutical applications.

## 2. Materials and Methods

### 2.1. Chemical Materials

HPLC-grade catechin (≥98% purity) was purchased from Sigma-Aldrich (St. Louis, MO, USA). Poly-D-glucosamine, glyceryl monostearate (GMS), caprylic triglyceride, sodium chloride (NaCl), sodium lauryl sulfate (SLS), potassium chloride (KCl), disodium phosphate (Na_2_HPO_4_), monopotassium phosphate (KH_2_PO_4_), bovine serum albumin (BSA), and thidiazuron (TDZ) were all analytical grade and purchased from Sigma-Aldrich (St. Louis, MO, USA). Ethoxylated sorbitan monooleate (Tween^®^ 80) and α-hydro-ω-hydroxypoly(oxyethylene) poly(oxypropylene) poly(oxyethylene) block copolymer (Poloxamer^®^ 188) were commercial grade and purchased from Sigma-Aldrich (St. Louis, MO, USA). Cell culture-grade Gamborg B5 medium including vitamins was purchased from Duchefa Biochemie BV (Haarlem, The Netherlands). High-performance liquid chromatography (HPLC)-grade acetonitrile was purchased from Merck (Darmstadt, Germany). Analytical-grade methanol, ethanol, and formic acid were purchased from RCI Labscan Ltd. (Dublin, Ireland).

### 2.2. V. coerulea Protocorm Methanolic Extract

*V. coerulea* callus was obtained from the Plant Tissue Culture Laboratory at Maejo University, Chiang Mai, Thailand. The fresh *V. coerulea* callus was subcultured in B5 liquid medium with 3 µM TDZ at 25 °C and 90 rpm under a 16 h photoperiod with a light intensity of 50 μmol/m^2^·s for four weeks. Then, 2 µM poly-D-glucosamine was applied, and the cultures were further incubated for another four weeks under the same conditions. The protocorms were then rinsed, dried at 50 °C for 2 days, and ground into powder, which was used for further extraction following the method outlined by Amnuaykan et al. (2024) [3]. Briefly, the *V. coerulea* protocorms were extracted by macerating 100 mg of dried powder in 10 mL of 80% *v*/*v* methanol for 12 h with continuous stirring. The extract was then sonicated, centrifuged, filtered through a 0.45 µm Millipore filter, and evaporated using a rotary evaporator (N-1001S-W, Eyela, Tokyo, Japan) set at 50 °C under 180 Pas to remove the methanol. The resulting methanolic extract was stored in a light-protected container at 4 °C until it was used for NLC development.

### 2.3. Phytochemical Analysis of V. coerulea Extract Using High-Performance Liquid Chromatography (HPLC) with Photodiode Array (PDA) Detection

Phytochemical analysis of *V. coerulea* protocorm methanolic extract was performed using HPLC based on our previously established method [3]. Briefly, the HPLC analysis was carried out using a Series 40 HPLC system (Shimadzu Corporation, Kyoto, Japan) equipped with a PDA detector (SPD-M40, Shimadzu Corporation, Kyoto, Japan). Separation was achieved using an Eclipse XDB-C18 column (150 mm × 4.6 mm i.d., 5 µm). The *V. coerulea* methanolic extract was dissolved in 50% *v*/*v* ethanol to obtain a final concentration of 2.5 mg/mL. Prior to injection, both the sample solutions and mobile phases were filtered through a 0.45 µm Millipore GV filter (Millipore, Bedford, MA, USA). A 10 µL aliquot of the sample solution was injected into the HPLC system. The gradient mobile phase consisted of acetonitrile (phase A) and 0.1% formic acid in DI water (phase B), with a flow rate of 1.0 mL/min. The elution program was as follows: 10% A for 10 min; increase to 20% A over 15 min (10–25 min); increase to 40% A over 5 min (25–30 min); further increase to 60% A over 5 min (30–35 min); and finally, return to 10% A over 5 min (35–40 min). Catechin, a major bioactive component of the *V. coerulea* methanolic extract, was used as a marker compound for quantification [3]. The PDA detector was set to scan in the wavelength range of 200–800 nm, and chromatograms were monitored at 280 nm for catechin detection. All analyses were performed in triplicate.

### 2.4. Fourier Transform Infrared (FT-IR) Spectroscopic Analysis of V. coerulea Extract

The molecular composition of the *V. coerulea* protocorm methanolic extract and catechin was analyzed using FT-IR spectroscopy. An aliquot of each sample was applied to a diamond Attenuated Total Reflectance (ATR) accessory of an Alpha II FT-IR spectrometer (Bruker, Karlsruhe, Germany). The infrared spectra were collected over the wavenumber range of 4000 to 400 cm^−1^, with transmittance plotted on the *Y*-axis and wavenumber (cm^−1^) on the *X*-axis.

### 2.5. Development of Blank NLCs

Blank NLC formulations were developed using the emulsification–ultrasonication method, wherein the aqueous phase is added to the lipid phase under agitation to form a coarse emulsion, followed by sonication to reduce the particle size and enhance stability [19]. Various factors affecting the NLC formulation were investigated, including the ratio of solid to liquid lipid, as well as the duration of ultrasonication. In brief, the NLCs were developed with a lipid content of 5% *w*/*w*, using GMS as the solid lipid and caprylic triglyceride as the liquid lipid at ratios of 9:1, 8:2, and 7:3. The combination of Poloxamer^®^ 188 and Tween^®^ 80 at a weight ratio of 2:1, resulting in a total concentration of 3% *w*/*w*, was used as the surfactant system. The lipid phase, consisting of GMS and caprylic triglyceride, was heated to 80 °C, while the aqueous phase, comprising Poloxamer^®^ 188, Tween^®^ 80, and deionized water, was prepared separately and heated to 85 °C. Following this, the aqueous phase was gradually added to the lipid phase and subjected to probe sonication using an ultrasonic processor (model VCX 600, Vibra Cell, Sonics & Materials, Newtown, CT, USA) set at 80 W, operating in a 10 s pulse-on and 2 s pulse-off cycle for 5 min. The NLCs were then kept in well-sealed containers until the characterization. The most favorable solid-to-liquid lipid ratio was selected to evaluate the ultrasonication duration, which was set to 5, 10, 20, and 30 min.

### 2.6. Characterization of Blank NLCs

Blank NLC formulations were characterized based on their physical appearance. In addition, their particle size, polydispersity index (PDI), and zeta potential were evaluated using a Zetasizer (Zetasizer^®^ version 5.00, Malvern Instruments Ltd., Malvern, UK). Prior to measurement, each NLC formulation was diluted 10,000-fold with DI water to avoid multiple scattering effects. The diluted samples were placed into disposable sizing cuvettes for particle size and PDI analysis, and into clear disposable zeta cells for zeta potential measurement. Hydrodynamic diameters corresponding to the Z-average values were reported. All measurements were performed in triplicate.

### 2.7. Stability Test of Blank NLCs

Blank NLC formulations were evaluated for their stability through both centrifugation and accelerated stability testing under heating–cooling conditions. The physical stability of the NLC formulations was evaluated using a centrifugation test [20,21]. Briefly, a 1 mL aliquot of each NLC formulation was transferred into individual centrifuge tubes and subjected to centrifugation using a microcentrifuge (MINI-10K+, Hangzhou Miu Instruments Co., Ltd., Hangzhou, China) set at 5000 rpm for 15 min [22]. Following centrifugation, the samples were visually examined for evidence of phase separation or sedimentation. The characteristics of the blank NLC formulations were also evaluated under accelerated heating–cooling conditions. Each blank NLC formulation was stored in a well-sealed glass bottle wrapped in aluminum foil for light protection and subjected to six cycles of alternating temperatures. Each cycle consisted of storage at 45 °C for 24 h, followed by storage at 4 °C for 24 h. After eight cycles, the physical appearance was observed, and particle size, PDI, and zeta potential were measured using a Zetasizer (Zetasizer^®^ version 5.00, Malvern Instruments Ltd., Malvern, UK). All measurements were performed in triplicate.

### 2.8. Development of NLC Containing V. coerulea Extract

The methanolic extract of *V. coerulea* protocorm was incorporated into the best NLC formulation, which was selected based on its favorable characteristics, including a small particle size, narrow PDI range, pronounced zeta potential, and stability after centrifugation as well as under accelerated heating–cooling cycling. The concentration of the *V. coerulea* protocorm extract ranged from 0.01% *w*/*w* (equivalent to 0.1 mg/mL) to 0.1% *w*/*w* (equivalent to 1 mg/mL), which can be considered effective, as these levels are substantially higher than the IC_50_ values previously reported for its DPPH inhibition, ferric-reducing, collagenase inhibition, elastase inhibition, and tyrosinase inhibition activities [3]. The extract was dissolved in the surfactant system comprising Poloxamer^®^ 188 and Tween^®^ 80. The resulting mixture was then added to the aqueous phase (preheated to 85 °C) and gradually incorporated into the lipid phase (maintained at 80 °C) while being subjected to probe sonication using an ultrasonic processor (model VCX 600, Vibra Cell, Sonics & Materials, Newtown, CT, USA) set at 80 W, operating in a 10 s pulse-on and 2 s pulse-off cycle for 5 min. The NLC containing the *V. coerulea* extract was evaluated based on its physical appearance, particle size, PDI, and zeta potential, as previously described in Section 2.5, and evaluated for both stability through both centrifugation and accelerated stability testing under heating–cooling conditions, as previously described in Section 2.6.

### 2.9. Determination of Entrapment Efficiency and Loading Capacity of V. coerulea Extract in NLCs

The entrapment efficiency and loading capacity of the *V. coerulea* extract in the NLCs were evaluated based on the method described by Chuesomboon et al. (2025), with slight modifications [23]. Briefly, NLCs containing the methanolic *V. coerulea* extract were centrifuged at 4000 rpm for 20 min. The resulting supernatant was collected and analyzed by HPLC, using catechin as a marker compound, to determine the amount of non-encapsulated extract. All measurements were conducted in triplicate. The encapsulation efficiency was calculated using the following equation: Encapsulation efficiency (% *w*/*w*) = (A − B)/A × 100, where A is the total content of *V. coerulea* extract that was initially incorporated and B is the amount of *V. coerulea* extract detected in the supernatant. The loading capacity was calculated using the following equation: Loading capacity (% *w*/*w*) = (A − B)/C × 100, where A is the total content of *V. coerulea* extract that was initially incorporated, B is the amount of *V. coerulea* extract detected in the supernatant, and C is the total weight of the NLC formulation. All analyses were performed in triplicate at 25 °C.

### 2.10. Investigation of Release Behavior of V. coerulea Extract from NLCs

The release profile of the *V. coerulea* extract from the nanostructured lipid carriers (NLCs) was evaluated using the dialysis membrane method following the method of Chaiyana et al. (2020) with minor modifications [24]. Dialysis bags (Cellu Sep T2, Membrane Filtration Products Inc., Frilabo, Maia, Portugal) were pretreated by boiling them in DI water for 15 min, followed by immersion in phosphate-buffered saline (PBS) (pH 5.5) for 24 h. Prior to the experiment, the dialysis bags were blotted dry with tissue paper, filled with 1 mL of the sample, and then sealed by tying them with wire to obtain a final size of 2.5 cm × 2.5 cm. Each dialysis bag was immersed in 50 mL of PBS (pH 5.5) containing 0.4% *w*/*v* BSA and maintained at 32 °C under continuous stirring using a magnetic hot plate stirrer (Velp Scientific Inc., Milan, Italy). These conditions (pH of 5.5 and temperature of 32 °C) were maintained to simulate the physiological environment of the skin surface [25]. The release of the *V. coerulea* extract at various time points (0, 0.5, 2, 8, and 24 h) was evaluated by sampling 5 mL of the medium, which was immediately replaced with an equal volume of fresh medium. The samples were analyzed by HPLC to quantify the released compounds following the method described in Section 2.3. The NLC containing the *V. coerulea* extract was compared with a hydroalcoholic solution of the *V. coerulea* extract, which was prepared using a 1:1 mixture of ethanol and DI water as the solvent. Blank formulations of both the NLC and the hydroalcoholic solution without the extract were also evaluated as controls. All analyses were performed in triplicate.

### 2.11. Investigation of the Skin Retention of V. coerulea Extract from NLCs

The skin retention of the *V. coerulea* extract from NLCs was evaluated using an ex vivo piglet skin model [26]. The premature stillborn piglets, which died of natural causes, were sourced from a local farm in Chiang Mai, Thailand, and collected shortly after their deaths. Ethical concerns regarding the use of animal skin in initial permeation studies can be alleviated when the skin is obtained from animals that have passed away naturally [27]. Full-thickness skin from the flank area of the piglet was used for the skin retention measurement. The hair was trimmed off, and the skin was carefully dissected into square pieces measuring 2 cm × 2 cm using a surgical blade. The subcutaneous fat layer was then removed with surgical scissors. Skin retention was measured in both the stratum corneum and the deeper skin layers, including the viable epidermis and dermis, which had a combined average thickness of 288.5 ± 66.2 µm. The skin was then rinsed with PBS (pH 5.5) and placed onto gauze pads pre-soaked with normal saline solution (NSS: 0.9% *w*/*v* NaCl) in Petri dishes. An additional 20 mL of NSS was added to keep the gauze moist throughout the investigation period. Subsequently, 100 µL of the sample was applied to the skin surface, and skin samples were collected after 1 h. The collected skin samples were washed with DI water to remove any surface residues and blotted dry with tissue paper. Subsequently, the skin was cut into small pieces and homogenized with 10 mL of 70% *v*/*v* ethanol using a high-performance dispersing instrument (T25 digital Ultra-Turrax^®^, IKA-Werke GmbH & Co. KG, Staufen, Germany) for 2 min. The resulting mixture was centrifuged using a Thermo Fisher Scientific centrifuge (Sorvall ST16R, Waltham, MA, USA) to separate the skin debris. The supernatant was collected and analyzed for *V. coerulea* extract content by HPLC following the method described in Section 2.3. The NLC containing 0.1% *w*/*w V. coerulea* extract was evaluated in comparison with a hydroalcoholic solution of the *V. coerulea* extract at the same concentration (0.1% *w*/*w*) that was prepared using a 1:1 mixture of ethanol and DI water as the solvent. Blank formulations of both the NLC and the hydroalcoholic solution without the extract were also evaluated as controls. All analyses were performed in triplicate.

### 2.12. Irritation Evaluation Using Hen’s Egg Test Chorioallantoic Membrane (HET-CAM) Assay

The irritation potential of the NLC containing 0.1% *w*/*w V. coerulea* protocorm extract and the solutions was assessed using the HET-CAM method [28]. Ethical approval is not required for the HET-CAM test since it employs fertilized hen eggs at an early embryonic stage, prior to the development of a functional nervous system capable of perceiving pain. It is a scientifically accepted alternative to conventional animal testing that aligns with ethical standards and regulatory efforts to reduce animal use while ensuring product safety [29,30]. Prior to the experiment, the chorioallantoic membrane (CAM) of fertilized eggs aged 7–9 days was prepared by carefully removing the eggshell and moistening the membrane with a 0.9% *w*/*v* sodium chloride solution. A 30 µL aliquot of each test sample was then applied directly onto the CAM surface. Irritation responses, including hemorrhage, vascular lysis, and coagulation, were observed for 5 min under a stereo microscope (Carl Zeiss™ Stemi 508 doc, Zeiss, Oberkochen, Germany). The onset times (in seconds) for hemorrhage (H), vascular lysis (L), and coagulation (C) were recorded and used to calculate the irritation score (IS) based on the following formula:

IS = [(301 − H) × 5]/300 + [(301 − L) × 7]/300 + [(301 − C) × 9]/300,(1)

If no irritation was observed within the 5 min, a score of zero was assigned for that endpoint. The resulting IS values were categorized as follows: 0.0–0.9 indicates no irritation, 1.0–4.9 indicates mild irritation, 5.0–8.9 indicates moderate irritation, and 9.0–21.0 indicates severe irritation. Sodium lauryl sulfate (1 mg/mL) and a normal saline solution were used as the positive and negative controls, respectively. The normal saline solution was also used to dissolve the *V. coerulea* protocorm extract at the same concentrations as in the NLC formulations. All experiments were conducted in duplicate.

### 2.13. Statistical Analysis

All data were expressed as the mean ± standard deviation (SD). Statistical differences among samples were analyzed using one-way analysis of variance (ANOVA) in GraphPad Prism (version 8.0.2; GraphPad Software, Boston, MA, USA), followed by Tukey’s post hoc test. A *p*-value less than 0.05 was considered statistically significant.

## 3. Results and Discussion

### 3.1. V. coerulea Extract and Its Phytochemical Constituents

**Figure 1:**
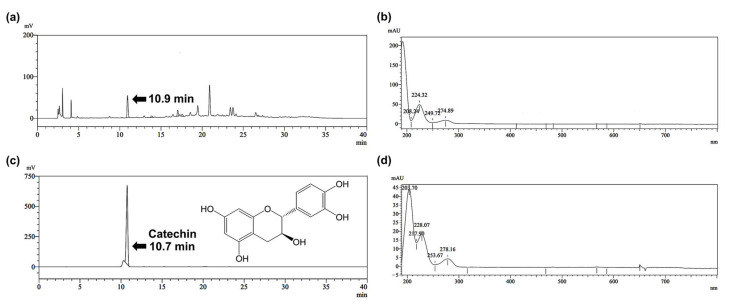
HPLC chromatogram of *V. coerulea* protocorm methanolic extract (**a**) and its corresponding photodiode array (PDA) spectrum (**b**), compared with the HPLC chromatogram of catechin standard (**c**) and its corresponding PDA spectrum (**d**), showing matching retention times and spectral characteristics.

*V. coerulea* protocorm was successfully extracted using 80% *v*/*v* methanol. The HPLC chromatograms shown in Figure 1 reveal multiple peaks in the extract chromatogram, indicating the presence of a complex mixture of phytochemicals. A major peak at approximately 10.7 min in the extract chromatogram closely matched the retention time of the catechin standard, indicating that catechin was a prominent component of the extract. The slight variation in retention time between the extract and the standard may be attributed to matrix effects or minor differences in chromatographic conditions. Due to the potential presence of compounds with comparable polarity or isomeric forms, the identification was verified by co-injecting the extract with a catechin standard. The catechin-spiked extract was used to confirm the identity of the peak corresponding to catechin in the sample. The findings are in line with a previous study that reported catechins as one of the flavonoids presented in high amounts in various orchids, including *Dendrobium nobile*, *Dendrobium moschatum*, *Dendrobium densiflorum*, and *Coelogyne nitida* [31]. To further support the identification, the PDA spectra of the peak were compared with that of the reference catechin compound. The findings revealed that the PDA spectrum of the 10.9 min peak in the extract displayed λ_max_ at approximately 224 and 274 nm, consistent with the spectrum of the catechin standard, which showed λ_max_ at around 228 and 278 nm. Minor absorbance features in both spectra were also highly comparable. The close agreement in both retention time and UV-Vis spectral characteristics between the *V. coerulea* protocorm extract and the catechin standard provides strong evidence for the presence of catechin in the protocorm extract.

**Figure 2:**
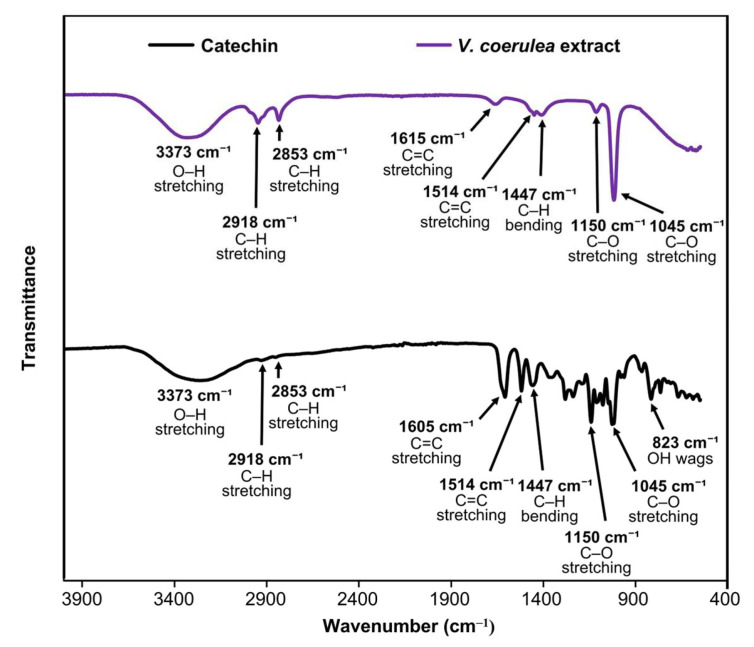
FTIR spectra of standard catechin (black) and *V. coerulea* protocorm methanolic extract (violet).

In addition, FTIR spectroscopy was used as a complementary technique to support the identification of catechin in the *V. coerulea* protocorm extract. FTIR spectra in the wavenumber range of 400 to 4000 cm^−1^ of the extract and standard catechin are presented in Figure 2, and the corresponding band assignments are summarized in Table 1. Both spectra display characteristic absorption bands that indicate the presence of similar functional groups, suggesting that catechin is a major component in the *V. coerulea* protocorm extract. The findings were consistent with previously reported data on catechin [32], further confirming the reliability of the observed spectral patterns and supporting the identification of catechin as a key constituent in the extract. A broad and intense absorption band at 3373 cm^−1^ in both spectra corresponds to O–H stretching vibrations, indicative of the presence of hydroxyl groups. This broad peak is characteristic of polyphenolic compounds such as catechins, known for extensive hydrogen bonding. The peaks at 2853 cm^−1^ and 2918 cm^−1^ are attributed to symmetric and asymmetric stretching vibrations of aliphatic C–H bonds, respectively. These bands are common in organic compounds containing alkyl chains and appear consistently in both catechin and the *V. coerulea* protocorm extract. Aromatic C=C stretching vibrations are observed at 1514 cm^−1^ in both samples, aligning well with the reported literature value of 1520 cm^−1^ [32]. Another aromatic C=C vibration appears at 1615 cm^−1^ in the extract and 1605 cm^−1^ in catechin, both consistent with the presence of aromatic ring structures typical of flavonoids. C–H bending vibrations are identified at 1447 cm^−1^, while peaks at 1150 cm^−1^ and 1045 cm^−1^ correspond to C–O stretching vibrations of aromatic alcohols and general alcohols, respectively. The presence of these bands further confirms the polyphenolic nature of both samples. A band at 823 cm^−1^, observed only in the catechin spectrum, is assigned to O–H wagging, typically found in the 900–750 cm^−1^ region. This feature may be overlapped or less prominent in the *V. coerulea* protocorm extract due to matrix complexity or lower catechin concentration. The consistent band positions, particularly in O–H stretching, aromatic C=C, and C–O regions, suggested that catechin contributed significantly to the phytochemical profile of the *V. coerulea* protocorm extract. Minor differences in peak intensity and position can be attributed to the complexity of the extract matrix and potential presence of other phenolic constituents.

### 3.2. Blank NLCs

**Figure 3:**
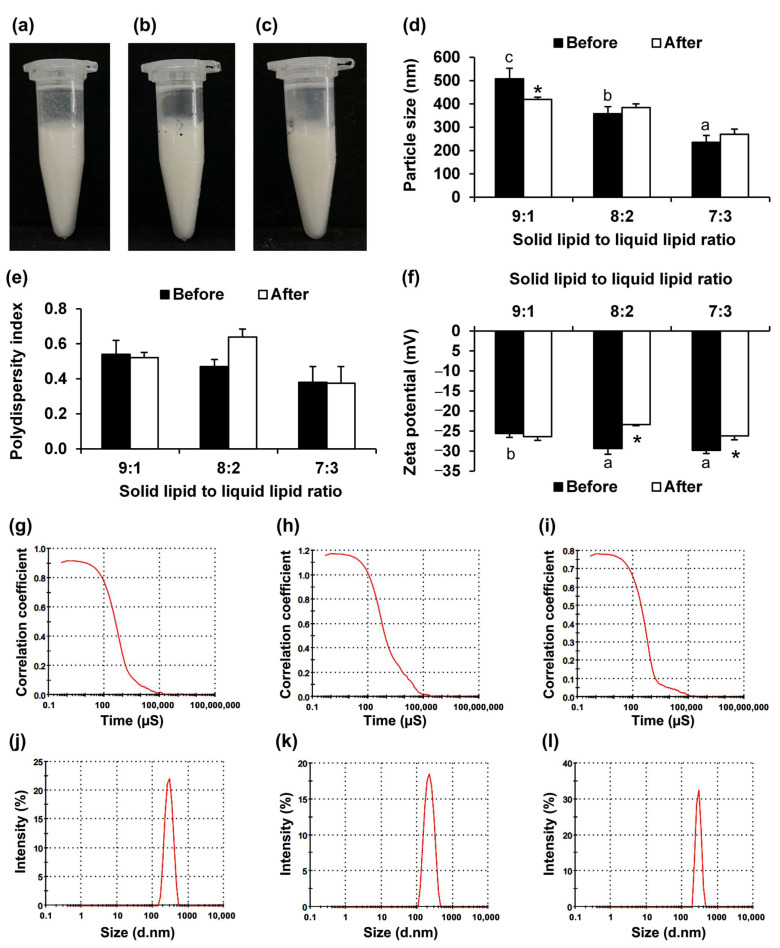
Effects of solid-to-liquid lipid ratio on NLC properties. Physical appearance of NLCs prepared with different solid-to-liquid lipid ratios of 9:1 (**a**), 8:2 (**b**), and 7:3 (**c**), along with their particle size (**d**), polydispersity index (**e**), and zeta potential (**f**). Dynamic light scattering (DLS) analysis showing correlation coefficient curves of NLCs prepared with solid-to-liquid lipid ratios of 9:1 (**g**), 8:2 (**h**), and 7:3 (**i**) and corresponding particle size distributions: 9:1 (**j**), 8:2 (**k**), and 7:3 (**l**). Different letters (a–c) indicate significant differences between samples, whereas asterisks (*) indicate significant differences in the same sample before and after accelerated stability test under heating–cooling cycling (*p* < 0.05).

The physical appearance of the NLCs prepared with varying solid-to-liquid lipid ratios (9:1, 8:2, and 7:3) is shown in Figure 3a–c. All the formulations appeared as homogeneous, opaque, milky white liquids with no visible changes in appearance after the stability test using centrifugation and accelerated stability test with eight heating–cooling cycles. These techniques are commonly used to evaluate the physical stability of cosmetic formulations [33]. Centrifugation subjects the formulations to high gravitational forces, thereby accelerating the potential separation of dispersed phases, which may occur more gradually under normal storage conditions. Similarly, cyclic temperature stress tests that alternate between 4 °C and 45 °C impose repeated thermal stress on the formulation, thereby facilitating the early detection of potential physical instabilities [33]. The use of 45 °C in the heating–cooling cycle was intended to simulate thermal stress conditions that may occur during storage and transportation. Storage at 45 °C was used as an accelerated stress condition for hot climatic zones (Zone IV) in accordance with international guidelines that recommend testing products at temperatures at least 15 °C above the intended storage temperature [34]. Although the solid-to-liquid lipid ratio did not significantly affect the physical appearance of the NLCs after exposure to the accelerated stress conditions, it influenced key formulation characteristics, including particle size, PDI, zeta potential, and stability, as shown in Figure 3d–f. The particle size of the NLCs significantly decreased with increasing liquid lipid content. The NLC formulation with a solid-to-liquid lipid ratio of 9:1 exhibited the largest particle size (507.9 ± 45.3 nm), whereas the 7:3 ratio resulted in the smallest particle size (235.5 ± 29.8 nm). This trend likely reflected the improved fluidity and dispersibility conferred by a higher proportion of liquid lipid, which facilitates the formation of smaller particles. These findings are consistent with those of previous studies that reported that higher levels of liquid lipid contribute to the formation of smaller NLC particles [35,36]. NLCs are considered oil-loaded SLNs that contain lipid droplets that are partially crystallized and have a less-ordered crystalline or amorphous solid structure; this finding by Müller et al. (2007) could be used to overcome the limitations of conventional SLNs [37]. At low oil concentrations, oil is molecularly dispersed within the lipid matrix, but exceeding its solubility limit induced phase separation and the formation of distinct oily nano-compartments [38]. The suggested ratio for solid lipids blended with liquid lipids to prepare the lipid matrix for NLCs typically ranges from 99.9:0.1 to 70:30 [38]. The findings from the current study also suggested that a ratio of 70:30 yielded the smallest NLC particle size. Aside from producing smaller NLC particles, the incorporation of liquid lipid in the NLC formulation also generated NLC particles with the oil embedded within the core of the solid lipid, resulting in a higher drug-loading capacity and controlled drug release since the drug is dissolved in the oil while simultaneously encapsulated by the solid lipid [38].

The dynamic light scattering analysis also provided further insights into the particle size characteristics and distribution of the NLCs prepared with varying solid-to-liquid lipid ratios. The correlation coefficient plots (Figure 3e–g) illustrate the decay rate of the correlation coefficient over time, reflecting the Brownian motion and uniformity of the nanoparticles. The NLC formulation with a 9:1 solid-to-liquid lipid ratio exhibited a slower decay, indicating larger particle sizes and greater heterogeneity. In contrast, the formulation with the solid-to-liquid lipid ratio of 8:2, and especially the 7:3 formulation, demonstrated a more rapid decay in the correlation curve, which is indicative of smaller and more uniformly distributed particles. These observations align with the average particle size results, where a significant decrease in particle size was observed with increasing liquid lipid content: 507.9 ± 45.3 nm in the 9:1 formulation to 235.5 ± 29.8 nm in the 7:3 formulation.

The solid-to-liquid lipid ratios also affected the stability of the NLC formulations. The particle size of the NLC with a solid-to-liquid lipid ratio of 9:1 significantly decreased from 507.9 ± 45.3 nm to 419.0 ± 9.9 nm after the heating–cooling cycling. Although a smaller particle size is generally preferable, the observed size reduction over time may have resulted from the leakage of liquid lipid from the NLC particles, indicating that the formulation may not be stable. However, increasing the ratio of liquid lipid led to greater stability, with no significant change in particle size observed over time. The findings were consistent with previous studies that noted that higher levels of liquid lipid contribute to the formation of more stable NLCs [35,36].

PDI, a parameter that describes the degree of non-uniformity in the particle size distribution of a sample, is a fundamental quality control parameter for lipid-based nanocarriers [39]. In drug delivery applications, especially those involving lipidic nanoparticles, assessing the particle size distribution is critical for ensuring formulation consistency, stability, and performance [40]. PDI values greater than 0.7 indicate a very broad and heterogeneous particle size distribution, which may compromise the reliability of dynamic light scattering measurements and often reflect formulation instability [37]. Conversely, a PDI of 0.3 or lower is typically regarded as acceptable, indicating a homogenous population of particles [41]. The solid-to-liquid lipid ratios did not have a significant effect on PDI (Figure 3e). However, a decreasing trend was observed as the liquid lipid ratio increased, with PDI values declining from 0.54 ± 0.08 to 0.38 ± 0.09 as the solid-to-liquid lipid ratio shifted from 9:1 to 7:3, hinting that the broader size distribution at higher solid lipid contents may result from increased crystallization and heterogeneity within the formulation. The relatively high PDI values indicate potential heterogeneity in the formulations, which may be attributed to the structural complexity of NLCs, particularly the coexistence of crystalline and amorphous regions [42]. However, the observed decrease in PDI with increasing liquid lipid content suggested that higher proportions of liquid lipid may reduce particle size variability by disrupting excessive crystallization due to its ability to form a less-ordered lipid matrix and promote a more homogeneous internal structure [43]. The particle size distributions (Figure 3h–j) support these findings. The formulation with a 9:1 solid-to-liquid lipid ratio exhibited a broader peak, suggesting a wider size range and potential polydispersity, whereas those with 8:2 and 7:3 ratios showed narrower and sharper peaks, indicating more monodisperse systems. No significant changes in PDI values were observed after the stability test, indicating that none of the NLC formulations underwent aggregation or significant broadening of their particle size distribution under stress conditions. Although the PDI values were higher than 0.3, they remained below 0.7, indicating a moderately uniform particle size distribution that is still acceptable for DLS analysis and suggests reasonable formulation stability. Although these results did not indicate instability under the test conditions, further optimization is needed to achieve a narrower size distribution and improved uniformity in the future.

Zeta potential plays a key role in the stability of nanosuspensions, with values exceeding ±30 mV generally considered necessary to achieve sufficient electrostatic repulsion and maintain particle stability [36]. The zeta potential measurements from the current study revealed that increasing the liquid lipid content enhanced the electrostatic stability of the formulations. The zeta potential values became progressively more negative with higher liquid lipid ratios, ranging from approximately −25.6 ± 1.0 mV for the 9:1 ratio formulation to −29.8 ± 0.8 for the 7:3 ratio formulation. This trend suggested stronger repulsive forces and greater colloidal stability at higher liquid lipid contents. However, after the stability test, a slight but statistically significant increase (i.e., less negative values) in zeta potential was observed for the formulations with the 8:2 and 7:3 ratios while the 9:1 formulation showed minimal changes. Despite this reduction, the values remained sufficiently negative (close to or above −25 mV), indicating that the formulations remained reasonably stable. Therefore, the NLCs with higher liquid lipid contents demonstrated better electrostatic stability and maintain relatively stable zeta potential values post-treatment, suggesting improved nanosuspension stability.

Thus, it could be concluded that increasing the liquid lipid proportion improves the physical stability of NLCs by reducing the particle size and maintaining a uniform distribution. The 7:3 solid-to-liquid lipid ratio formulation exhibited the most favorable characteristics, including the smallest particle size, a narrow PDI range, and adequate zeta potential after the stability testing, making it a promising formulation for stable NLC production. Additionally, the dynamic light scattering correlation and intensity profiles confirmed that increasing the proportion of liquid lipid enhanced particle uniformity and reduced the average particle size.

**Figure 4:**
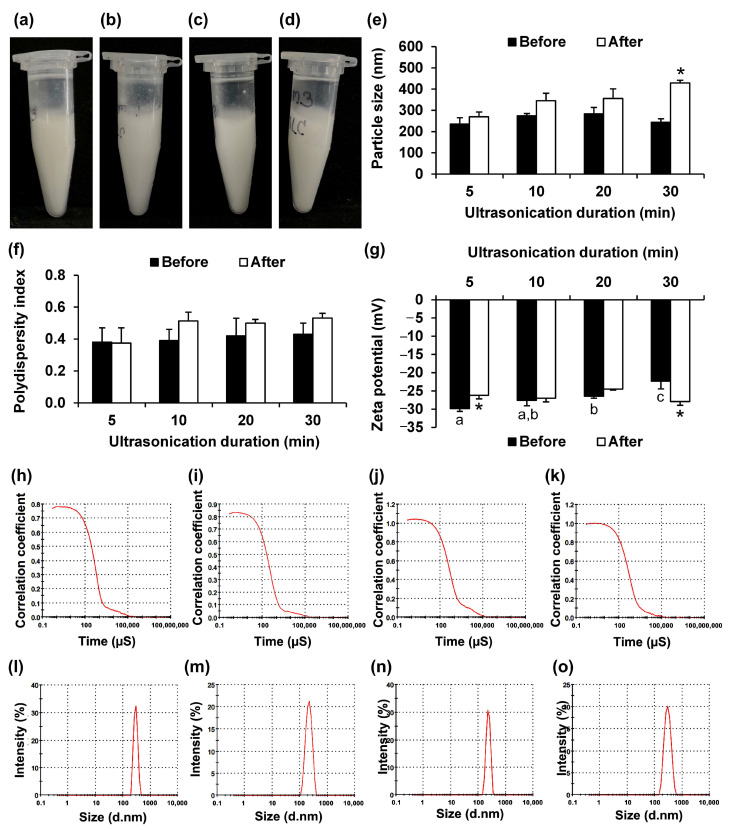
Effects of ultrasonication duration on NLCs. Physical appearance of NLCs prepared with ultrasonication durations of 5 min (**a**), 10 min (**b**), 20 min (**c**), and 30 min (**d**), along with their particle size (**e**), polydispersity index (**f**), and zeta potential (**g**). Dynamic light scattering (DLS) analysis showing correlation coefficient curves of NLCs prepared with ultrasonication durations of 5 min (**h**), 10 min (**i**), 20 min (**j**), and 30 min (**k**) and corresponding particle size distributions: 5 min (**l**), 10 min (**m**), 20 min (**n**), and 30 min (**o**). Different letters (a–c) indicate significant differences between samples, whereas asterisks (*) indicate significant differences in the same sample before and after accelerated stability test under heating–cooling cycling (*p* < 0.05).

On the other hand, the influence of ultrasonication duration on the physical characteristics and stability of the blank NLCs was investigated by evaluating their visual appearance, particle size, PDI, and zeta potential (Figure 4). The visual appearances of the NLCs prepared using various ultrasonication durations were similar: a homogeneous, opaque, milky liquid that remained stable after centrifugation and accelerated stability studies under heating and cooling conditions. Furthermore, no significant differences were observed in particle size and PDI. Across all ultrasonication durations, the correlation coefficient curves exhibited sharp transitions, indicating relatively monodisperse particle systems. The size distribution graphs further corroborated the particle size trends, as each ultrasonication duration yielded a single, prominent peak, reflecting a relatively uniform particle size distribution. The NLCs sonicated for 5 min exhibited the smallest and most stable particle size, remaining relatively unchanged after storage. In contrast, prolonged sonication for 30 min resulted in a significant increase in particle size after storage (*p* < 0.05), indicating instability and aggregation. This trend may be attributed to the thermal effects of prolonged bath sonication, which can cause partial melting of the lipid matrix (GMS and caprylic triglyceride) and promote polymorphic transitions. Yajima et al. (2002) demonstrated that 50 °C is the optimum temperature for transforming GMS from the metastable α-form to the more thermodynamically stable β-form, which packs more tightly and forms larger crystals [44]. In contrast, a shorter ultrasonication time followed by rapid cooling helped to preserve a more disordered or mixed polymorphic state, leading to the formation of smaller and more stable NLCs. Additionally, extended sonication could induce the degradation of the surfactant structure and stabilize the NLC particles. Li et al. (2019) reported that the prolonged and high-intensity ultrasonication broke down polysaccharides into shorter chains, reducing their emulsifying capacity and leading to phase separation in O/W emulsions during storage [45]. These phenomena could also occur with the surfactant molecules (Poloxamer^®^ 188 and Tween^®^ 80) used in the current study and result in the observed increase in particle size at longer ultrasonication times. Although no significant differences were observed among the NLCs with different ultrasonication durations, a slight increase in PDI was noted as the sonication time increased from 5 to 30 min. This suggested that prolonged sonication may also lead to a broader particle size distribution, likely due to the formation of aggregates.

In contrast to the effects on particle size and PDI, the zeta potential values changed, becoming more negative with longer ultrasonication durations, further confirming the destabilizing effect of excessive sonication. The sample sonicated for 5 min exhibited the most negative zeta potential value (−27.6 ± 1.47 mV). In contrast, the zeta potential of the sample treated for 30 min was significantly less negative (−22.3 ± 2.1 mV), indicating reduced surface charge stability. This reduction in surface charge suggested diminished electrostatic repulsion among particles, increasing the likelihood of aggregation. The weakened zeta potential might result from degradation or inactivation of surfactant molecules caused by prolonged ultrasonication and thermal stress, leading to poor interfacial stabilization.

Therefore, optimizing the ultrasonication duration is crucial in the formulation of NLCs to maintain the desired physicochemical properties and achieve storage stability. The findings from the current study indicated that shorter sonication durations (5 min) were more favorable for producing stable, small NLCs with a narrow size distribution and high surface charge. The NLCs prepared using 5 min of ultrasonication, with a total lipid content of 5% *w*/*w*, a GMS-to-caprylic triglyceride ratio of 7:3, and a surfactant blend of Poloxamer^®^ 188 and Tween^®^ 80 at a 2:1 weight ratio (totaling 3% *w*/*w*) were selected for further incorporation of the *V. coerulea* extract. In future studies, transmission electron microscopy or scanning electron microscopy imaging could be performed to provide detailed visual information on particle shape and surface characteristics, which would enhance the comprehensiveness of the NLC formulation analysis.

### 3.3. NLC Containing V. coerulea Extract

**Figure 5:**
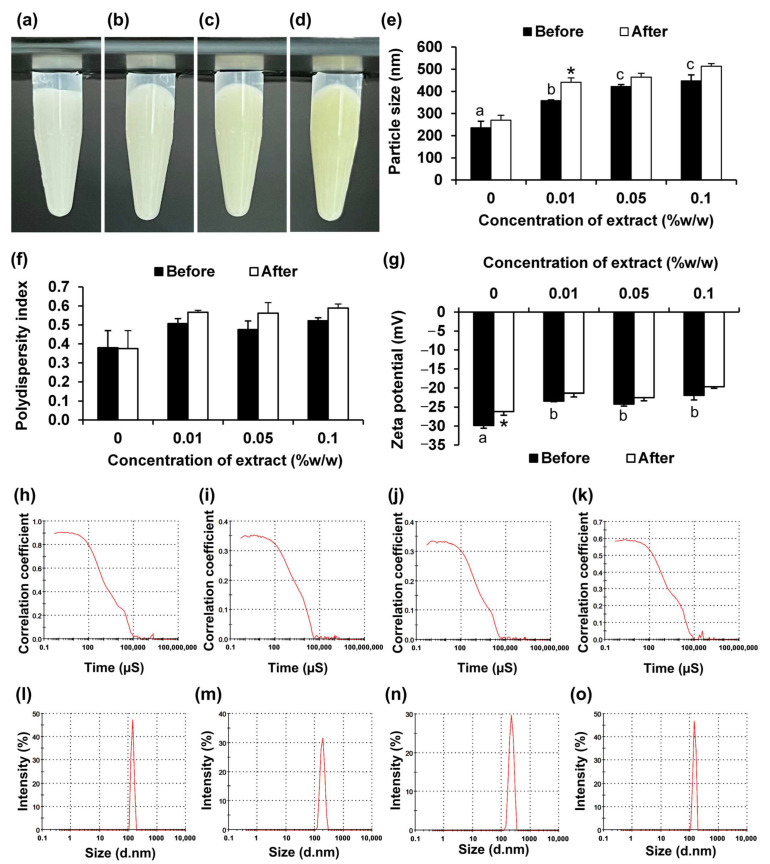
Effects of *V. coerulea* extract on NLCs. Physical appearance of blank NLC (**a**) and the NLC containing various concentrations of *V. coerulea* extract, including 0.01% *w*/*w* (**b**), 0.05% *w*/*w* (**c**), and 0.1% *w*/*w* (**d**), along with their particle size (**e**), polydispersity index (**f**), and zeta potential (**g**). Dynamic light scattering (DLS) analysis showing correlation coefficient curves of blank NLC (**h**) and the NLC containing various concentrations of *V. coerulea* extract of 0.01% *w*/*w* (**i**), 0.05% *w*/*w* (**j**), and 0.1% *w*/*w* (**k**), and corresponding particle size distributions: blank (**l**), 0.01% *w*/*w* (**m**), 0.05% *w*/*w* (**n**), and 0.1% *w*/*w* (**o**). Different letters (a–c) indicate significant differences between samples, whereas asterisks (*) indicate significant differences in the same sample before and after accelerated stability test under heating–cooling conditions (*p* < 0.05).

The methanolic extract of chitosan-treated *V. coerulea* protocorms, which has been shown to exhibit potent collagenase inhibition activity, anti-tyrosinase activity, strong anti-inflammatory effects, and no signs of irritation or cytotoxicity [3], was incorporated into the developed NLCs to overcome the skin penetration limitations associated with its hydrophilic nature. Various concentrations of the *V. coerulea* extract ranging from 0.01 to 0.1% *w*/*w*, which are considered effective since they exceeded the IC_50_ values previously reported for its DPPH-radical scavenging, ferric-reducing antioxidant, and collagenase, elastase, and tyrosinase inhibition activities [3]. The incorporation of increasing concentrations of the extract into the NLCs influenced their physical characteristics and stability, as shown in Figure 5. Visually, all formulations remained homogeneous, although a slight yellowish hue was observed at higher extract concentrations, especially at 0.1% *w*/*w*. Additionally, the NLC particle size increased significantly with the extract incorporation and continued to grow larger as the concentration increased. This enlargement of the NLC particles suggests that the incorporated extract was successfully encapsulated into the lipid matrix, leading to an increase in particle size.

In terms of PDI, no effect was observed after incorporating the extract. In contrast, the zeta potential became less negative with increasing extract content, dropping from −29.8 ± 1.0 mV in the blank NLC to −21.9 ± 1.2 mV at 0.1% *w*/*w*. This reduction in surface charge may be attributed to the presence of neutral or slightly polar compounds in the extract that adsorbed onto the particle surface, diminishing the electrostatic repulsion and potentially compromising colloidal stability over time. Despite these changes, no visible phase separation occurred after storage, indicating that the NLC system maintained acceptable physical stability across all extract concentrations tested.

Additionally, the correlation coefficient curves indicated that the blank NLC exhibited a sharp and consistent decay, reflecting monodisperse particles. With the incorporation of 0.01% *w*/*w* extract, the decay remained sharp, suggesting minimal disruption to particle homogeneity. However, higher extract concentrations (0.05% and 0.1% *w*/*w*) resulted in broader, less defined curves, indicating increased polydispersity. These trends aligned with the particle size distribution results, where the blank NLC showed a narrow peak with a small particle size and low PDI. Incorporation of 0.01% extract slightly increased the size, but it maintained a narrow distribution, while higher concentrations led to broader peaks and larger particles. The increase in particle size and PDI at higher extract levels may be attributed to interactions between the extract and lipid matrix.

Although the findings noted that increasing the extract concentration affected the NLC particle size, resulting in larger particles and a less pronounced PDI, these values remained within the acceptable range, and the NLC containing the highest concentration of *V. coerulea* protocorm extract (0.1% *w*/*w*) was used in the subsequent evaluations, as higher concentrations of the extract could potentially provide greater benefits.

### 3.4. Entrapment Efficiency and Loading Capacity of NLC Containing V. coerulea Extract

The entrapment efficiency and loading efficacy of the NLC containing 0.1% *w*/*w V. coerulea* protocorm extract were found to be 75.0 ± 4.2% and 0.5 ± 0.1%, respectively. The relatively high entrapment efficiency indicates that the active compound was effectively encapsulated within the lipid matrix of the NLCs, suggesting good compatibility between the extract and the lipid components. This efficient encapsulation is crucial for enhancing the stability of the bioactive compounds, which is particularly advantageous for cosmetic applications, as it can facilitate the effective encapsulation of extracts within the lipid core, thereby protecting them from environmental degradation [46,47]. By embedding the extract within the lipid core, NLCs could significantly extend the shelf life and preserve the biological activities of the formulation. In contrast, the relatively low loading capacity may be attributed to the limited concentration of the *V. coerulea* protocorm extract, which was only used at a concentration of 0.1% *w*/*w*. Consequently, when compared with the overall composition of the NLC system, the resulting loading capacity was relatively low at 0.5 ± 0.1% *w*/*w*. In addition, the limited solubility or saturation potential of the extract within the lipid phase may present a common limitation in lipid-based delivery systems, particularly when encapsulating hydrophilic plant-derived compounds. While the low loading capacity implied that only a small proportion of the total formulation consisted of active compounds, this limitation did not undermine the effectiveness of the delivery system. These findings are consistent with previous studies reporting that curcumin-loaded NLCs achieved a high entrapment efficiency of 93.01, whereas the loading capacity was relatively low at 0.80% [48]. Additionally, high entrapment efficiencies of various plant-derived extracts within lipid-based delivery systems have been widely reported. Encapsulation efficiencies of a polyphenol-rich Hibiscus sabdariffa extract ranged from 52.9 ± 0.9% to 93 ± 3% *w*/*w* for quercetin and from 60 ± 2% to 84 ± 4% *w*/*w* for anthocyanins [49]. Similarly, an *Ocimum sanctum* Linn. extract achieved an entrapment efficiency of 87.4 ± 5.6% *w*/*w* in NLCs [24], while hesperetin was encapsulated with an efficiency of 72.7 ± 0.92% [50].

Since the *V. coerulea* protocorm extract is a complex mixture of natural bioactive components, it could have varying polarities and molecular characteristics. This diversity may lead to selective entrapment, where certain constituents preferentially incorporate into the lipid matrix of NLCs based on their physicochemical compatibility. As such, the EE and LC determined in the current study, using catechin as a marker compound, may not fully represent the encapsulation of all bioactive constituents within the extract. However, catechin was chosen due to its known bioactivity and quantifiability, serving as a suitable representative to estimate the overall encapsulation performance of the system [3]. It is important to note that the actual EE and LC for the entire extract likely reflect the averaged behavior of multiple constituents, some of which may be more or less efficiently encapsulated depending on their affinity for the lipid components. However, as catechin was noted as a bioactive marker, the high entrapment efficiency achieved in the current study suggested that the developed NLC formulation has significant potential in cosmetic applications. Further investigations into its in vitro release kinetics and skin permeation behavior are essential to confirm the delivery efficiency and functional performance of the formulation.

### 3.5. Release Behavior of V. coerulea Extract from NLCs

**Figure 6:**
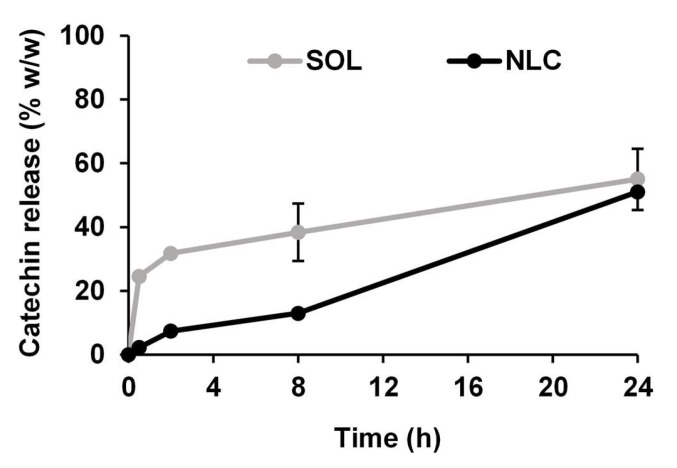
Release profile of *V. coerulea*-extract-loaded NLC (NLC) compared to hydroethanolic *V. coerulea* extract solution (SOL).

The in vitro release profiles of the *V. coerulea*-extract-loaded NLCs and the hydroethanolic *V. coerulea* extract solution were compared (Figure 6). Catechin, the major bioactive component of the extract, was used as a marker compound for the quantitative analysis of the extract. The catechin release from the hydroethanolic solution was significantly higher than that from the NLC at all time points, reaching approximately 55.05 ± 9.62% *w*/*w* at 24 h, whereas the NLC only released 51.06 ± 0.56% *w*/*w*. The hydroethanolic solution exhibited an initial burst of release within the first 2 h, indicative of immediate diffusion of free catechins. In contrast, the NLC formulation demonstrated a sustained release pattern, with a slower and more controlled release rate over time. This behavior can be attributed to the encapsulation of the *V. coerulea* extract within the lipid matrix, which serves as a barrier, limiting rapid diffusion [51]. In topical delivery systems, a sustained-release profile offers several advantages. It prolongs the residence time of active compounds on the skin, helping to maintain effective local concentrations and reducing the need for frequent reapplication [52,53]. Moreover, a controlled release enhances skin absorption and minimizes the risk of irritation often associated with an initial burst release [54,55]. Catechin, a key bioactive compound in the extract, is known to be sensitive to oxidation [56]. The encapsulation of catechin within the NLC system likely provides protection against oxidative degradation, thereby preserving its bioactivity during storage and application. Aside from catechin, various other bioactive components are also present in the *V. coerulea* protocorm extract [3], each potentially contributing to the overall therapeutic activity and exhibiting distinct entrapment and release behaviors depending on their physicochemical properties. The release behavior of natural extracts from NLCs can be inherently complex due to the multicomponent nature of the extract. While catechin was used as a marker to assess in vitro release, it might not capture the full spectrum of release dynamics for all components. Some highly lipophilic constituents may remain associated with the lipid core longer, resulting in a sustained release, whereas more hydrophilic components might diffuse out more readily [57,58]. Nevertheless, using catechin as a bioactive marker, the findings support the potential of NLCs as effective delivery systems for topical applications, offering sustained release, which may prolong the biological activities of bioactive compounds on the skin and enhance their functional performance [52]. Further investigations using pure catechin as a reference compound incorporated into the NLCs could provide additional insights into its individual behavior compared to that within a complex extract matrix.

### 3.6. Skin Retention of V. coerulea Extract from NLCs

**Figure 7:**
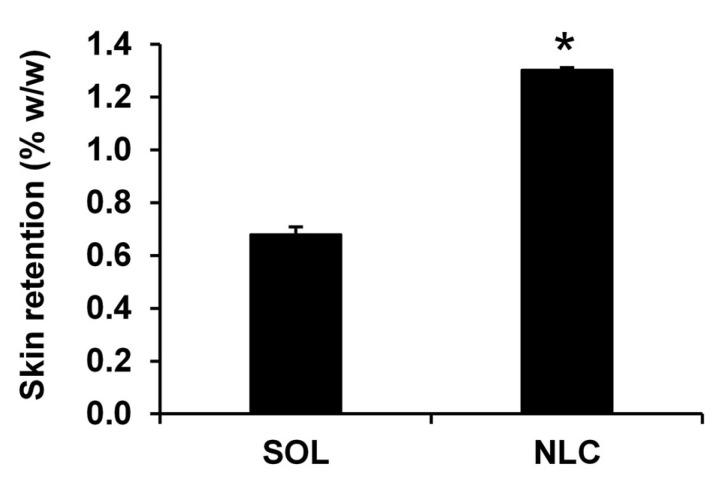
Skin retention of *V. coerulea* extract-loaded NLC (NLC) compared to hydroethanolic *V. coerulea* extract solution (SOL). Asterisk (*) denotes a significant difference in skin retention between formulations (*p* < 0.05).

In the current study, the NLC was designed to enhance the delivery of the bioactive constituents of the *V. coerulea* protocorm extract into the skin layers. The goal was to target the epidermis and dermis layers with no systemic absorption to enhance efficacy and ensure safety in compliance with regulatory standards for topical cosmetics. The skin retention of the *V. coerulea* extract-loaded NLC and that of the hydroethanolic *V. coerulea* extract solution after 1 h of application were compared, and the results indicated that the NLC formulation achieved significantly higher skin retention than the solution (Figure 7). After 1 h, the *V. coerulea*-extract-loaded NLC exhibited approximately twice the retention compared to the hydroethanolic solution. The concentration of catechin in the extracted media from skin treated with the NLC containing the *V. coerulea* protocorm extract for 1 h was 2.18 ± 0.01 mg/mL, corresponding to 1.30 ± 0.01% *w*/*w*, while that of the hydroethanolic solution was 1.89 ± 0.02 mg/mL, corresponding to 0.68 ± 0.03% *w*/*w*. The statistically significant difference indicated the enhanced ability of NLCs to retain the bioactive compounds in the *V. coerulea* extract in the skin (*p* < 0.05). This enhanced skin retention can be attributed to the occlusive properties and nanoscale characteristics of the NLC system, which promote closer interaction with the skin surface and facilitate greater accumulation of bioactive compounds within the stratum corneum [59]. Additionally, the sustained release profile of the NLC system likely contributed to the prolonged contact time with the skin surface, further improving bioactive accumulation. Additionally, the NLC system developed in this study is composed of GMS and caprylic triglyceride, both of which are structurally similar to lipids naturally present in the skin barrier [60]. This compositional similarity may enhance the affinity of the formulation for the stratum corneum, thereby promoting greater skin retention and improved dermal delivery of the active compounds. In summary, the significantly improved skin retention observed with the *V. coerulea* extract-loaded NLC was attributed to its nanoscale structure, sustained release behavior, and biomimetic lipid composition, all of which contributed to its demonstrated potential for use in topical antioxidant and anti-aging cosmetic applications.

### 3.7. Irritation Properties of NLC Containing V. coerulea Extract

**Figure 8:**
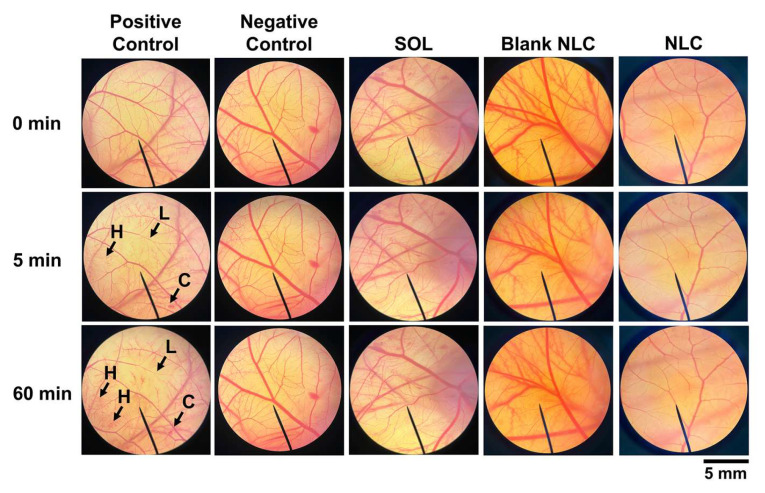
Microscopic images (magnification: 10×) showing the effect of the positive control (1% *w*/*v* sodium lauryl sulfate solution), negative control (0.9% *w*/*v* NaCl solution), 0.1% *w*/*w V. coerulea* extract in 0.9% *w*/*v* NaCl solution, blank NLC, and NLC containing 0.1% *w*/*w V. coerulea* extract on the chorioallantoic membrane after 0, 5, and 60 min of the exposure. H represents hemorrhage, L represents vascular lysis, and C represents coagulation.

The irritation scores of the NLC containing the *V. coerulea* extract and its corresponding solution are presented in Table 2, while representative images of the CAMs exposed to each formulation are shown in Figure 8. The positive control triggered immediate irritation, leading to hemorrhage, vascular lysis, and coagulation. It exhibited severe irritation with an irritation score of 9.1 ± 1.8. In contrast, the negative control caused no irritation on the CAM even after 60 min of exposure (IS = 0.0 ± 0.0). These findings support the suitability of the HET-CAM assay for predicting irritation potential, as it successfully differentiated between the non-irritating negative control and the severely irritating positive control. The current study noted that the *V. coerulea* extract did not elicit any vascular responses (hemorrhage, lysis, or coagulation) on the CAM, indicating that the extract itself is non-irritating at the tested concentration of 0.1% *w*/*w*. Similarly, the blank NLC formulation, which only contained the NLC base without the extract, showed no signs of irritation, suggesting that the carrier system was biocompatible and safe for topical application. Importantly, the NLC formulation containing 0.1% *w*/*w V. coerulea* extract also demonstrated no irritation on the CAM. This indicated that the incorporation of the extract into the NLC system did not alter its irritation profile. The absence of irritation in both the *V. coerulea* extract solution and NLC formulation confirmed the non-irritating nature of the *V. coerulea* extract and supported the use of the NLC as a safe delivery system for this botanical ingredient. These findings align with the intended application of the NLC formulation in dermatological or cosmetic products, where safety and skin tolerance are critical considerations. Furthermore, the consistency of these results reinforces the reliability of the HET-CAM assay in predicting irritation potential for both hydrophilic solutions and lipid-based nanocarriers.

## 4. Conclusions

The NLCs developed in the present study successfully encapsulated the catechin-rich *V. coerulea* extract, demonstrating strong potential for cosmetic applications. The optimized formulation, composed of GMS, caprylic triglyceride, Poloxamer^®^ 188, and Tween^®^ 80, along with the *V. coerulea* extract, exhibited favorable physicochemical properties, including good stability, high entrapment efficiency, a sustained release profile, and enhanced short-term skin retention. These findings support the potential use of this NLC system as an effective carrier for antioxidant-rich plant extracts in topical skincare formulations. As long-term stability data for NLCs during storage are essential for evaluating the shelf life and suitability of the formulation for practical applications, future studies should incorporate both physical and chemical stability assessments (e.g., measurement of catechin content) over extended storage periods. Additionally, future studies could incorporate the nanoparticle-loaded system into a topical gel since this type of formulation could provide additional advantages, including improved skin adhesion, controlled spreading, enhanced patient compliance, and prolonged residence time at the site of application.

## Figures and Tables

**Table 1:** Characteristic bands on FTIR spectra of *V. coerulea* protocorm extract compared with the catechin standard and literature values.

Band Assignment	Wavenumber (cm^−1^)		
	*V. coerulea* Extract	Catechin	
		Standard	Literature [32]
O–H wags	Overlapping region	823	900–750
–C–O strech	1045	1045	1015
–C–O aromatic alcohol	1150	1150	1190
–C–O alcohol	1447	1447	1280
C=C aromatic ring	1514	1514	1520
C=C aromatic ring	1615	1605	1618
Asymmetric aliphatic C–H bonds	2853	2853	–
Symmetric aliphatic C–H bonds	2918	2918	–
O–H stretching	3373	3373	3350

**Table 2:** Irritation severity scores of NLC containing *V. coerulea* extract and its corresponding solution.

Samples	Irritation Score	Severity
Positive control	9.1 ± 1.8 ^a^	Severe irritation
Negative control	0.0 ± 0.0 ^b^	No irritation
SOL	0.0 ± 0.0 ^b^	No irritation
Blank NLC	0.0 ± 0.0 ^b^	No irritation
NLC	0.0 ± 0.0 ^b^	No irritation

Note: Positive control = 1% *w*/*v* sodium lauryl sulfate solution; negative control = 0.9% *w*/*v* NaCl solution; SOL = 0.1% *w*/*w V. coerulea* extract in 0.9% *w*/*v* NaCl solution; Blank NLC = NLC base without *V. coerulea* extract; NLC = NLC containing 0.1% *w*/*w V. coerulea* extract. Different letters (a and b) indicate significant differences in irritation scores between samples (*p* < 0.05).

## Data Availability

The data supporting the findings of this study are included in this article.

## Abbreviations

The following abbreviations are used in this manuscript:

ANOVA	Analysis Of Variance
BSA	Bovine Serum Albumin
CAM	Chorioallantoic Membrane
GMS	Glyceryl Monostearate
HPLC	High-Performance Liquid Chromatography
HET-CAM	Hen’s Egg Test on Ahorioallantoic Membrane
IS	Irritation Score
NaCl	Sodium Chloride
NLC	Nanostructured Lipid Carrier
NSS	Normal Saline Solution
PBS	Phosphate-Buffered Saline
PDI	Polydispersity Index
KCl	Potassium Chloride
SD	Standard Deviation
SLNs	Solid Lipid Nanoparticles
TDZ	Thidiazuron
